# The role of hypoxia and radiation in developing a CTCs-like phenotype in murine osteosarcoma cells

**DOI:** 10.3389/fcell.2023.1222809

**Published:** 2023-11-16

**Authors:** Martina Quartieri, Anggraeini Puspitasari, Tamara Vitacchio, Marco Durante, Walter Tinganelli

**Affiliations:** ^1^ Biophysics Department, GSI Helmholtzzentrum für Schwerionenforschung GmbH, Darmstadt, Germany; ^2^ Biology Division, Gunma University Heavy Ion Medical Center, Maebashi, Japan; ^3^ Institut für Festkörperphysik, Technische Universität Darmstadt, Darmstadt, Germany

**Keywords:** tumor metastasis, cancer stem cells, circulating tumor cells, tumor hypoxia, radiation

## Abstract

**Introduction:** Cancer treatment has evolved significantly, yet concerns about tumor recurrence and metastasis persist. Within the dynamic tumor microenvironment, a subpopulation of mesenchymal tumor cells, known as Circulating Cancer Stem Cells (CCSCs), express markers like CD133, TrkB, and CD47, making them radioresistant and pivotal to metastasis. Hypoxia intensifies their stemness, complicating their identification in the bloodstream. This study investigates the interplay of acute and chronic hypoxia and radiation exposure in selecting and characterizing cells with a CCSC-like phenotype.

**Methods:** LM8 murine osteosarcoma cells were cultured and subjected to normoxic (21% O2) and hypoxic (1% O2) conditions. We employed Sphere Formation and Migration Assays, Western Blot analysis, CD133 Cell Sorting, and CD133+ Fluorescent Activated Cell Sorting (FACS) analysis with a focus on TrkB antibody to assess the effects of acute and chronic hypoxia, along with radiation exposure.

**Results:** Our findings demonstrate that the combination of radiation and acute hypoxia enhances stemness, while chronic hypoxia imparts a cancer stem-like phenotype in murine osteosarcoma cells, marked by increased migration and upregulation of CCSC markers, particularly TrkB and CD47. These insights offer a comprehensive understanding of the interactions between radiation, hypoxia, and cellular responses in the context of cancer treatment.

**Discussion:** This study elucidates the complex interplay among radiation, hypoxia, and cellular responses, offering valuable insights into the intricacies and potential advancements in cancer treatment.

## Graphical Abstract

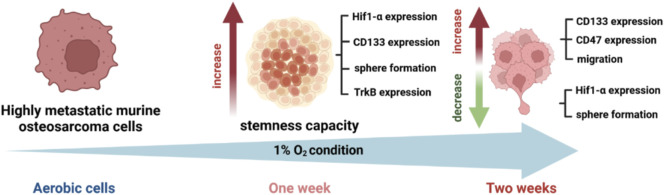

## 1 Introduction

Osteosarcoma is a highly heterogeneous malignant tumor, representing the most common primary tumor in bone (Kashima et al., 2003). Despite improvements in clinical treatment, osteosarcoma remains an aggressive tumor with a poor prognosis in the advanced stages. Metastasis are the primary cause of cancer morbidity and mortality, accounting for about 90% of cancer deaths (Gao et al., 2017). Osteosarcoma occurs in more than 80% of patients and usually metastasizes to the lungs and bones (De Luca et al., 2018), with a high probability of recurrence.

Not all tumor cells can form metastasis; the ones that trigger the process are cells that possess stem-like properties, also known as Cancer Stem Cells (CSCs) (Ayob and Ramasamy, 2018). This subset of cells is the key driver of tumor growth, recurrence, metastasis, and treatment resistance and can be identified by specific markers, like CD133 (Yang et al., 2015). Also known as, prominin-1, CD133 is a transmembrane glycoprotein involved in cell migration and cancer initiation (Tinganelli and Durante, 2020). It is well documented in literature that high protein levels of CD133 are related to high metastatic capability of cancer cells as well as an increased resistance to radiotherapy treatments (Liou, 2019).

Metastasis consists of a multi-step process, also known as ‘‘invasion-metastasis cascade’’ (Giancotti, 2013). First, metastatic cancer cells go through an Epithelial-to-Mesenchymal transition (EMT). The EMT phenotype decreases the cells’ adhesion, disaggregating them from the primary tumor, and increases their motility (Guarino et al., 2007). EMT contributes to the production of CSCs, initiating cells that shed from primary tumor sites (He et al., 2018), they migrate and intravasate into the blood or lymph circulation, becoming Circulating Tumor Cells (CTCs) (Park et al., 2020). CTCs can move inside of the blood circulation not only as a single cell but also as cell clusters (Hong et al., 2016; Krol et al., 2021; Chen et al., 2022). The clustering of CTCs shows an increased aggressiveness and metastatic capability, as well as an improved stemness capacity compared to single CTCs (Lin et al., 2021a; Schuster et al., 2021). As a result of the maintenance of cell-cell adhesion properties, cluster CTCs can be resistant to apoptosis; furthermore, they gain the ability to evade from the immune system detection (Leone et al., 2018; Amintas et al., 2020; Sun et al., 2022). In general, cancer cells in the bloodstream are subjected to stress and are destined to die due to a programmed cell death called ‘‘*anoikis*’’ (Guarino et al., 2007). By depriving the cells of adhesion signals necessary for cancer cell progression, *anoikis* acts as a physiological barrier to cancer progression (Geiger and Peeper, 2005). CTCs are able to resist *anoikis*, thus facilitating their spread and secondary tumor formation at distant sites (Douma et al., 2004).

*Anoikis* resistance might be conferred by specific cellular markers. TrkB is a neurotrophic tyrosine kinase receptor that has an oncogenic effect, according to literature. When overexpressed, it protects cells against programmed cell death, allowing them to survive in foreign environments even without physiologic adhesion signals (Geiger and Peeper, 2005). Inside the blood circulation, tumor cells are targeted and eliminated by the immune system. However, CTCs are able to evade the immune system detection by expressing a high amount of the “do not eat me” protein CD47 (Lian et al., 2019a). Among the cells that can survive in the bloodstream, only a small fraction of them are actually responsible for metastasis formation: these cell bear cancer stem cells characteristics such as stemness capacity and high invasiveness, and are called Circulating Cancer Stem Cells (Kantara et al., 2015; Yang et al., 2015; Luo et al., 2018). These cells are capable of undergoing a new phenotypic shift, known as a Mesenchymal-to-Epithelial transition (MET). This transition eventually allows them to form metastatic deposits at distant sites (Banyard and Bielenberg, 2015). Despite the available knowledge, the mechanisms underlying how certain cells can migrate and later form metastasis are still poorly understood.

A crucial role in the EMT transition, as well as in the enrichment of CSCs and CTCs formation is played by hypoxia (He et al., 2018). The normal level of oxygen in healthy tissues varies between ∼4.6% O_2_ to 9.4% O_2_ (Physoxia) (McKeown, 2014). In contrast, while the oxygen concentration in tumors can drop to 2% or even below (Muz et al., 2015). Hypoxia effects are modulated by Hypoxia-inducible factors (HIFs), composed by 3 different subunits which are important mediators in cellular adaptation to low oxygen (Ziello et al., 2007; Semenza, 2012; Tong et al., 2018). This evidence indicates that HIFs promote the stemness properties of Cancer Stem Cells and contribute to their maintenance in hypoxic niches (Yun and Lin, 2014; Vadde et al., 2017; Zhang et al., 2021), by expanding the subpopulation of cells positive for CD133 marker (Heddleston et al., 2010; Maeda et al., 2016) and increasing the expression of other stem cell markers such as Oct4 and Nanog (Lin et al., 2021b).

Indeed, oxygen deprivation is not the only stressor that might be involved in metastasis formation. Exogenous stressors such as radiation therapy could also play a role (Sundahl et al., 2018; Kim et al., 2019). Photon radiation could damage the vasculature in the primary tumor, leading to the release of CTCs into circulation (Koonce et al., 2017). This process selects Circulating Cancer Stem Cells due to their increased radioresistance compared to normal tumor cells (Schulz et al., 2019) and enhances the expression of matrix metalloproteinases (MMPs), which degrade the extracellular matrix, thereby increasing cell migration and invasion (Lee et al., 2017).

In the current study, we conduct *in vitro* experiments using stressors such as acute and chronic hypoxia, as well as photon radiation (X-rays), to select cells with Circulating Cancer Stem Cell (CCSCs)-like phenotype.

## 2 Materials and methods

### 2.1 Cell culture and culture condition

**FIGURE 1:**
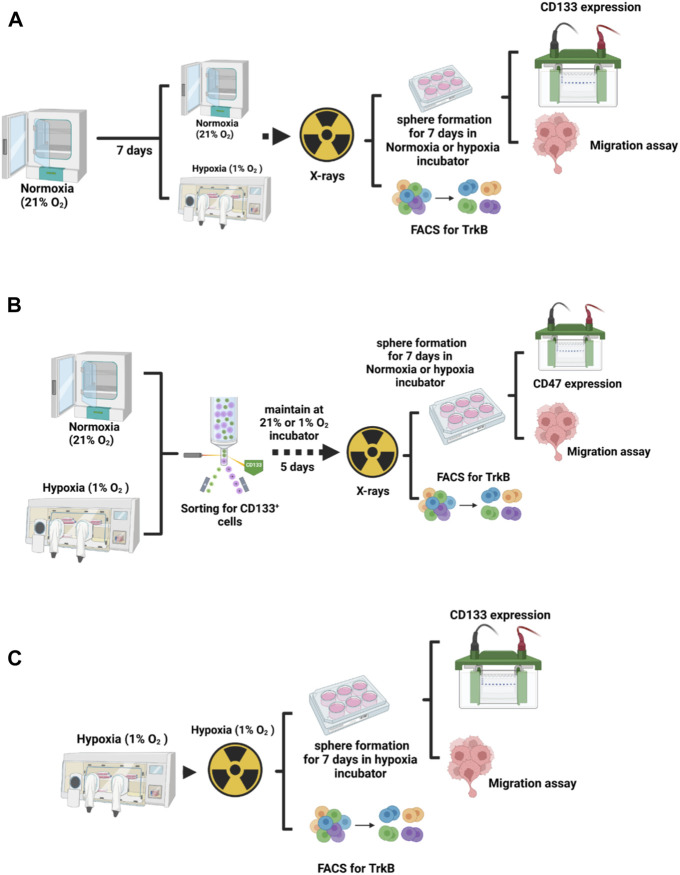
Experimental design. **(A)** To analyze the influence of hypoxia duration on the LM8 cell phenotype, cells were incubated in normoxia and hypoxia for one and 2 weeks, and then irradiated before proceeding with further study. **(B)** For the selection of cancer stem cells, LM8 cells were maintained under normoxic conditions, while hypoxic cells were kept inside a hypoxic working station (*In VivO*_2_ 400) for 1 week and 2 weeks. Subsequently, they were sorted for CD133-positive cells. After 5 days of incubation in normoxic or hypoxia conditions, cells were irradiated, and further analysis was performed. **(C)** LM8 cells were cultivated in hypoxia and irradiated under hypoxic conditions before further analysis.

We used the highly metastatic murine osteosarcoma cell line LM8 (Cell Bank Riken BioResource Research Center). Cells were cultured in Dulbecco’s Modified Eagle Medium (DMEM 1X + GlutaMAX-I), Gibco, Bleiswijk, Netherlands), supplemented with 10% fetal bovine serum (FBS superior, Sigma, Brazil) and 1% Penicillin-Streptomycin (10,000 U/mL Penicillin, 10 mg/mL Streptomycin, Pan Biotech, Aidenbach, Germany), in a humidified 5% CO_2_ incubator at 37°C. For the normoxic condition, we used 21% O_2_ (95% N_2_, 1L of H_2_O for humidification and 5% CO_2_ composition). For the hypoxic condition, cells were cultured in T75 tissue culture flasks inside of a hypoxic working station *In VivO*_2_ 400 (Baker Ruskinn, United Kingdom). The incubator setup included 94% N_2_, 1% O_2_ and 5% CO_2_, with 65% humidity, maintained at 37°C (Figures 1A–C). Cells were exposed to hypoxia for a week (Acute hypoxia) and 2 weeks (Chronic hypoxia), and always handled inside of the hypoxic working station, were washed using Dulbecco’s Phosphate Buffered Saline (Sigma Aldrich, United Kingdom), trypsinized with 1 mL of Trypsin-EDTA (0.05%/0.02%, Pan Biotech, Aidenbach, Germany) and reseeded at a 1:20 dilution.

### 2.2 Sphere formation and migration assay

LM8 were initially cultured under normoxic conditions for 1 week. Afterwards, they were irradiated with a dose of 4 Gy X-rays, counted with a cell counter (Beckman Coulter, United States), and then seeded at a density of 30.000 cells per well in ultra-low attachment 6-well plates (Greiner Bio-one, Germany). Following this, the plates were incubated under two different oxygen conditions: 21% O_2_ and 1% O_2_ (Acute Hypoxia) for an additional week, allowing the cells to form spheres in suspension. In parallel, LM8 were maintained under 1% O_2_ condition for 1 week, irradiated, seeded in the plates and kept for an additional week under 1% O_2_ condition (Chronic Hypoxia) (Figures 1A–C). Afterwards, sphere formation efficiency was considered according to the equation:

$$Sphere\;formation\;efficiency\;\left(\%\right)=\frac{Number\;of\;spheres}{Total\;number\;of\;cells}\times 100$$

In order to investigate the migration capacity, the spheres were embedded in collagen (Collagen I, rat tail, Enzo Life Sciences) and pictures (×4 magnification) were taken after 24 h and 48 h using a Motic AE31 microscope (camera: Moticam 3 + 3.0 MP; ocular lenses: WFPL ×10; objective lenses: ×4/0.10 ∞/- WD 23.5). Analysis was performed according to the following formula:

Area of migration=External area of migration−Internal area of the sphere

The area and the number of spheres in the clusters have been counted by segmenting the circumference of each sphere using ImageJ 1.53t software (Java 1.8.0_345 64-bit version).

### 2.3 Western blot

The cells were irradiated with 4 Gy X-ray and subsequently maintained in T75 flasks and ultra-low attachment 6-well plates at 21% O_2_ and 1% O_2,_ respectively. After 1 week, the cells were harvested on ice using 200 uL of RIPA-buffer (see Table 1). Sham controls were prepared for comparison. The scraped cells were transferred to 1.5 mL Eppendorf tubes and incubated for 30 min on ice, followed by centrifugation at 4°C for 15 min at 14.000 rpm. The supernatant was collected for protein quantification and further analysis. Protein quantification was performed using the DC protein Assay Kit from BioRad (Bio-Rad DC Protein Assay Kit 2, United States, N° 5000112). Equal amounts of protein (2.5–5 g per lane) were subjected to SDS-PAGE and transferred to Immobilon-P polyvinylidene difluoride membranes (Merck Millipore). The membranes were incubated with the appropriate primary and secondary antibodies, as listed in Tables 2, 3. Peroxidase activity was detected using chemiluminescence reagents (Pierce ECL Western blotting Substrate, Thermo Scientific, Rockford, United States), and visualized with an image analyzer (Fusion FX Vilber Lourmat, Peqlab) equipped with Fusion software.

**TABLE 1:** Details of RIPA.

1 × RIPA
1 × Halt Protease Inhibitor
1 mM Na-Orthovanadat
2 mM Na-Fluorid
ddH_2_O to bring to the volume needed

**TABLE 2:** List of primary antibodies used.

CD 133, Prominin 1 antibody, 1:1000, Abcam, RRID:AB_470302. CD47 Polyclonal Antibody, 1:1000, Invitrogen, RRID: AB_2899620
Monoclonal Anti-β-Actin Antibody, 1:10000, Sigma-Aldrich, RRID:AB_476744

**TABLE 3:** List of secondary antibodies used.

Goat Anti-Mouse IgG (H L)-HRP Conjugate antibody 1:3000, Bio-Rad, RRID:AB_11125547
Goat Anti-Rabbit IgG (H L)-HRP Conjugate antibody, 1:3000, Bio-Rad, RRID:AB_11125142

### 2.4 CD133 cell sorting

LM8 were cultured in T75 flasks for 1 week, in 21% O_2_ and 1% O_2,_ and subsequently harvested in Accutase (Sigma-Aldrich, St. Louis, United States) and centrifuged at 100g for 5 min (Figure 1B). Supernatant was discarded and cells were re-suspended in 1.5 mL of sterile PBS (Sigma Aldrich, United Kingdom) and then incubated with CD133 antibody (abcam ab19898) 1:200 dilution on a rotor for 1 h. Cells were then washed with PBS and incubated with 3 µL of Alexa Fluor 488 goat anti rabbit IgG for another hour. Cells were then filtered using a 5 mL Polystyrene Tube with Cell-Strainer Cap (Falcon 352235), and then sorted using a cell sorter (S3e Cell Sorter, Bio-Rad, Germany). After sorting, CD133+ cells were then plated in T75 flasks, kept for 5 days in the incubator to grow and then irradiated with 4 Gy X-rays. After irradiation, cells were harvested for Fluorescent Activated Cell Sorting (FACS) analysis.

### 2.5 CD133+ Fluorescent Activated Cell Sorting (FACS) analysis

Sorted cells were harvested with Accutase, washed with PBS, and incubated with antibody (Table 4) for 30 min at room temperature, in the dark. After, cells were washed and re-suspended in 100 µL of PBS, and measured using a Flow Cytometer (CytoFLEX, Beckman Coulter, United States). Data analysis was performed with the CytExpert v2.5 software. Gating strategy for the analysis was performed using a Viability Dye (VivaFix 649/660, Biorad) to discriminate between living and dead cells, and only viable cells were considered for the analysis. TrkB antibody (CF405M, Biorbyt) was used, and the filter considered was Pacific Blue 450V, with a 405 nm of wavelength.

**TABLE 4:** Antibodies used for FACS analysis.

TrkB antibody, 1:50, Biorbyt, United Kingdom
Viability Dye VivaFix 649/660, Biorad, US.

### 2.6 Statistical analysis

Statistical analysis was performed using the unpaired *t*-test. Values of *p* < 0.05 were considered statistically significant. Statistical analysis was performed to compare both normoxia and hypoxia, as well as controls and irradiated samples. All analyses were performed using ImageJ 1.53t software (Java 1.8.0_345 64-bit version), QuPath software (0.3.0 version), and GraphPad Prism 9 software.

## 3 Results

### 3.1 Hypoxia increases CD133 expression in LM8 cells

**FIGURE 2:**
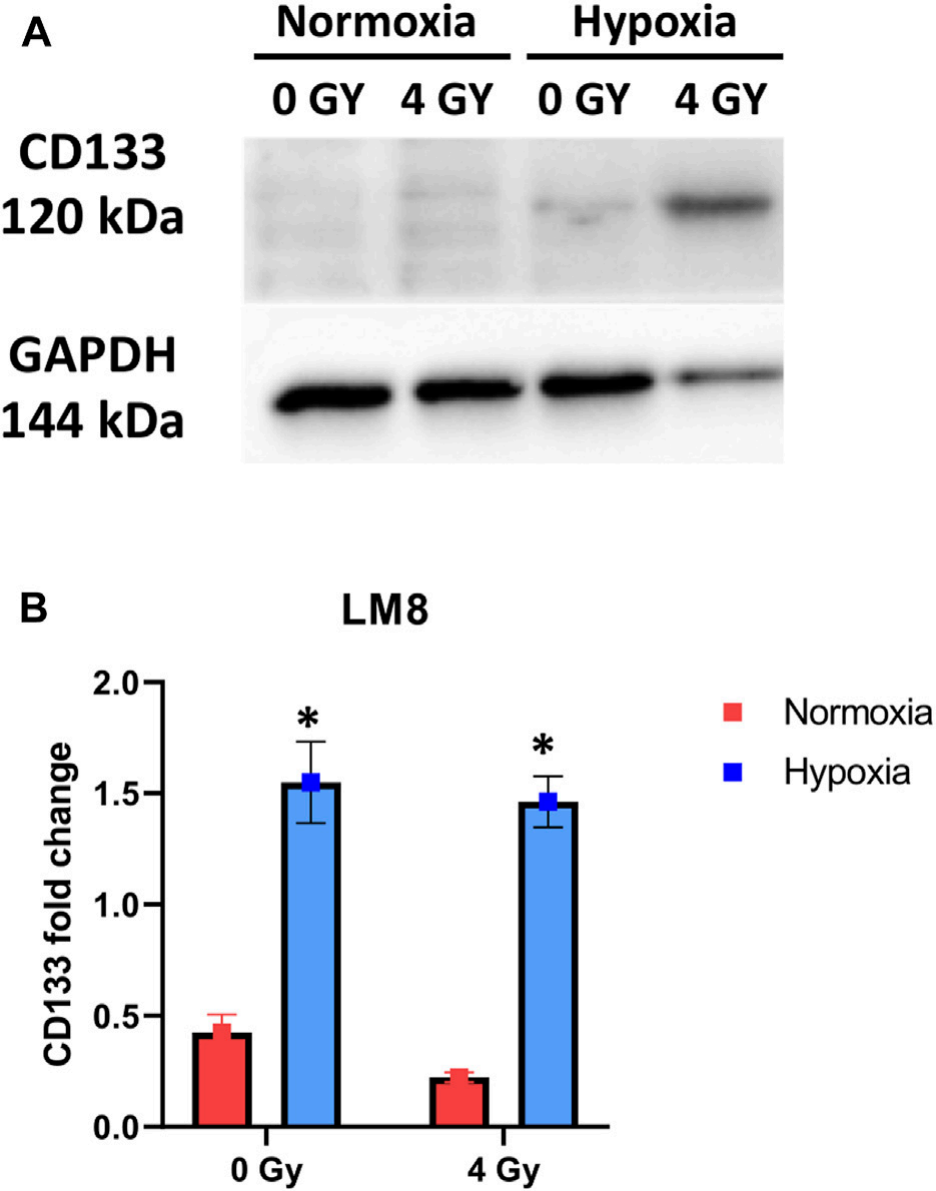
Cells were exposed to 1% O_2_ for 1 week. After the indicated time points, the cells were processed, and the total cell content of CD133 was detected through immunoblot analysis, with GAPDH serving as a loading control. A comparison was made between Normoxia and Hypoxia conditions, with 0 Gy as the control and 4 Gy as the irradiation dose **(A)** Representative experiment of Western blot. **(B)** Displays of the mean densitometry of CD133 relative to GAPDH, expressed in arbitrary units. *Hypoxic cells vs. normoxic control cells. ± SEM, *p* < 0.05, unpaired Student’s t-test.

In our study, we used murine osteosarcoma cell line LM8, a highly metastatic cell line when injected subcutaneously in C3H mice (Tanaka et al., 2013; Tinganelli et al., 2022). To investigate the hypoxic status of the cells, we analysed the expression of the HIF1a protein after 1 week and 2 weeks of hypoxic exposition. In prostate cancer, it has been shown that HIF1a protein levels decrease with prolonged exposure to hypoxia (Ravenna et al., 2014). In our experiments, the cells exposed to 1 week of hypoxia showed a substantial increase of HIF1a, confirming that the transition to chronic hypoxia and subsequent activation of genes, responsible for the cellular phenotype typical of aggressive and invasive tumors, had not yet occurred, while after 2 weeks of hypoxia, HIF1a level decreased (Supplementary Figure S1A). Therefore, we refer to the 1-week hypoxia as ‘‘Acute hypoxia’’ and the 2-week hypoxia as ‘‘Chronic hypoxia”. Under normoxia, the LM8 cells express CD133 with a fold change of approximately 0.5. Following hypoxia, CD133 significantly increases (*p*-value: 0.0049) and remains unaffected by photon radiation, with a fold change around 1.5 (Figures 2A, B).

### 3.2 Hypoxia increases the subpopulation of TrkB cells

**FIGURE 3:**
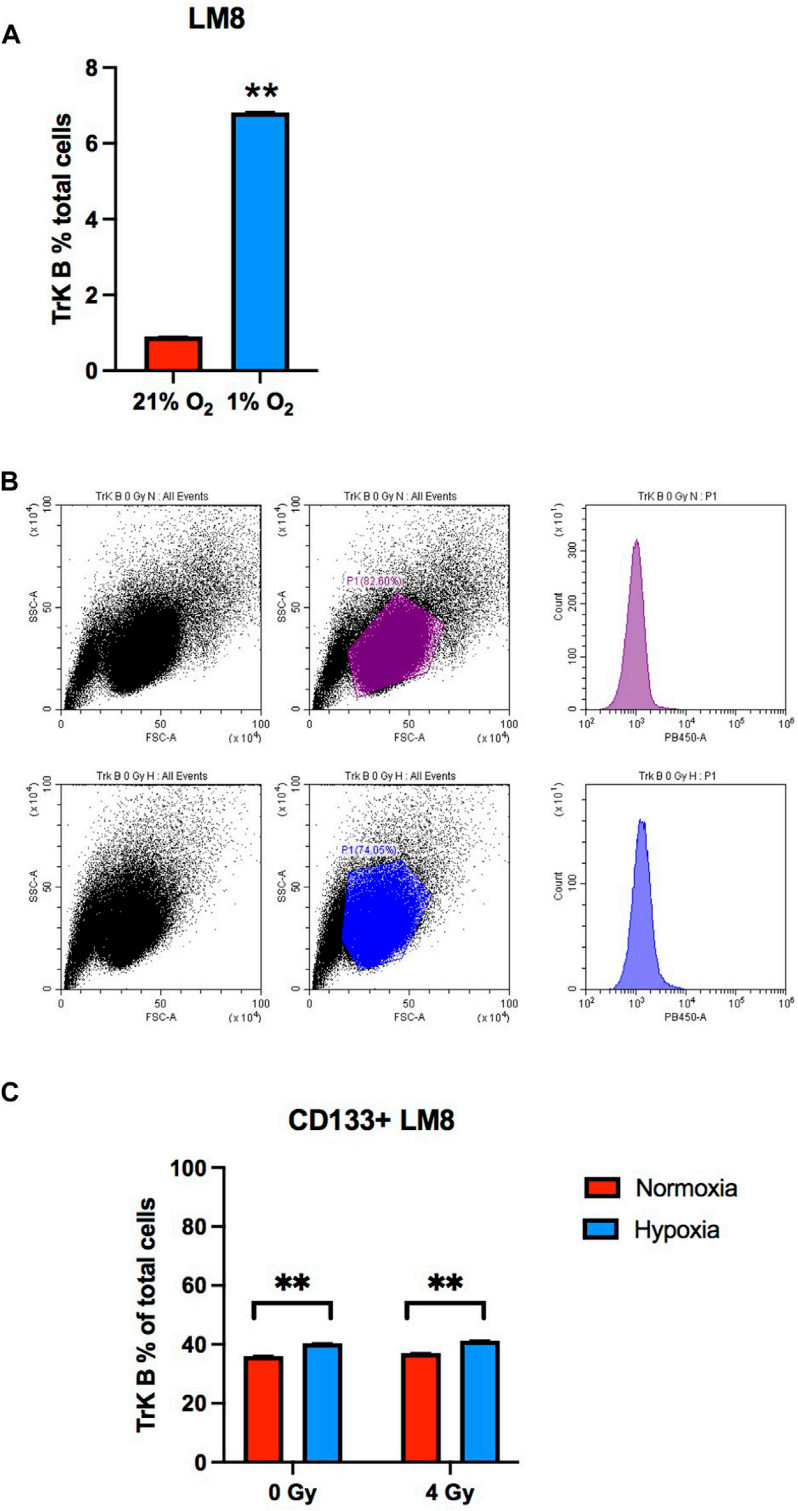
The TrkB expression on LM8 cells. **(A)** The expression of TrkB increased in hypoxic cells compared to normoxic cells **(B)**. Representative bar plots show the surface marker expression of TrkB on LM8 cells: the upper panel is from normoxic cells, and the lower panel is from hypoxic cells. **(C)** The expression of TrkB on CD133-positive cells was not influenced by hypoxia or radiation. A comparison was made between normoxia and hypoxia, considering 0 Gy as the control and 4 Gy as irradiation dose. * Hypoxic cells vs. normoxic control cells. ± SEM, *p* < 0.01.

CD133 is a marker for cell stemness, but CTCs also express other crucial markers, like the TrkB, that allow them to resist *anoikis* (Paoli et al., 2013; Adeshakin et al., 2021). In our study, we found that the TrkB percentage was around 1% in whole LM8 cells population. Under hypoxia, TrkB expression increased to around 7% (Figures 3A, B). Furthermore, to study the percentage of CD133+ cells that also express the TrkB marker, we sorted LM8 cells based on CD133+. In the sorted cells, TrkB expression significantly increases up to 40% (*p*-value: 0.0001) (Figure 3C).

### 3.3 Hypoxia and radiation changes sphere formation efficiency, sphere size and sphere clusters of LM8 cells

**FIGURE 4:**
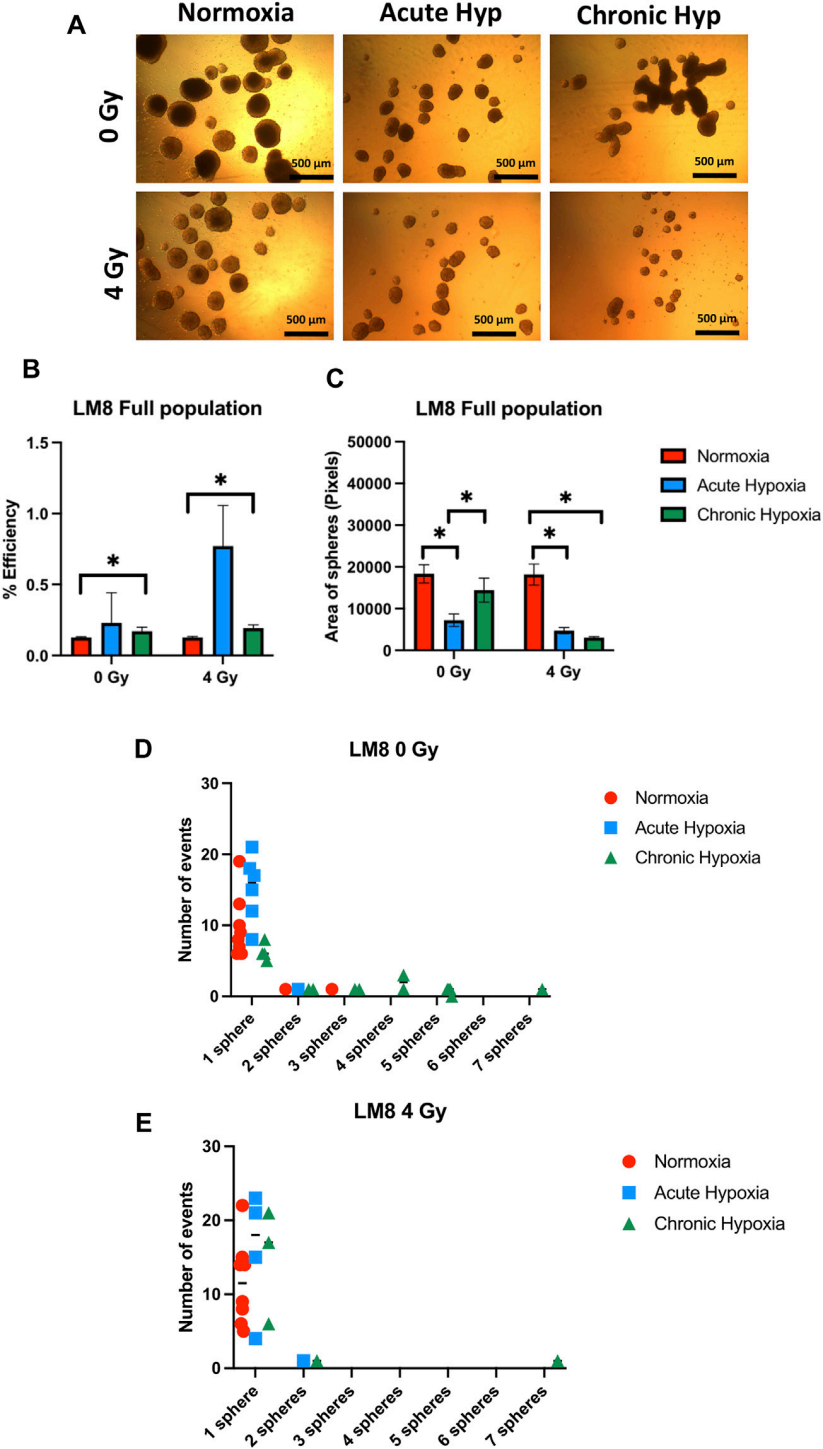
The effects of hypoxia and radiation on sphere formation. **(A)** Representative images of spheres. A comparison was made between normoxia, acute hypoxia and chronic hypoxia, with 0 Gy as the control and 4 Gy as the irradiation dose. ×4 magnification **(B)** Graphics of sphere efficiency formation, **(C)** Size of the sphere area (in pixels). **(D–E)** The number of events of aggregating spheres. Hypoxic cells vs. normoxic control cells ±SEM, *p* < 0.05, unpaired Student’s t-test.

**FIGURE 5:**
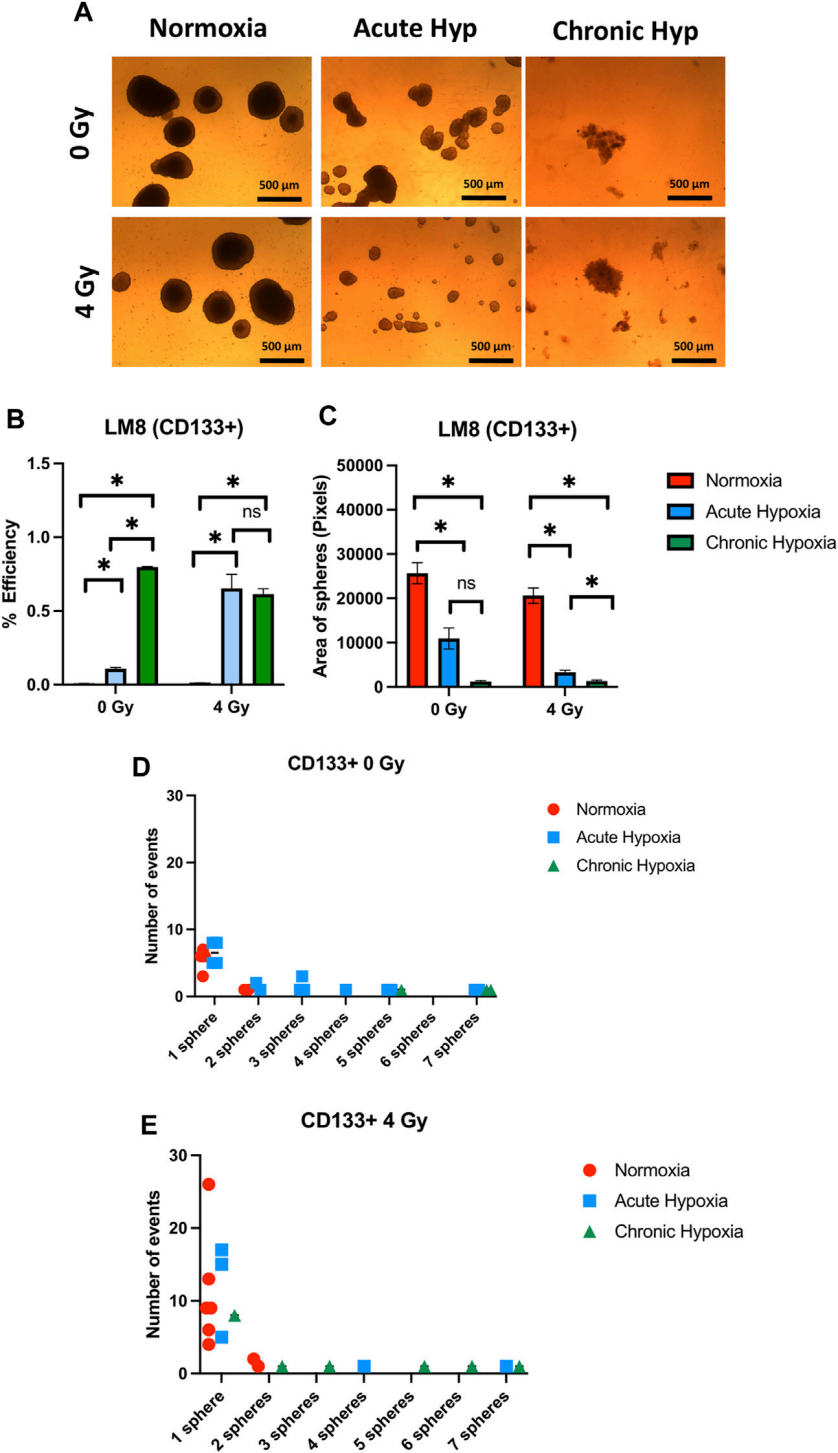
The effects of hypoxia and radiation on sphere formation of CD133 sorted LM8 cells. Comparison was made between normoxia, acute hypoxia and chronic hypoxia, with 0 Gy as the control and 4 Gy as the irradiation dose. **(A)** Representative images of spheres. **(B)** Graphs of sphere efficiency formation, **(C)** Size of the sphere area (in pixels). **(D–E)** The number of events of aggregating spheres. Hypoxic cells vs. normoxic control cells ±SEM, *p* < 0.05, unpaired Student’s t-test.

The duration of exposure to hypoxia strongly influences the ability to form spheres, and the size of spheres in the entire population of LM8 cells. Under acute hypoxia, LM8 cells show increased spheres formation efficiency (from 0.12% to 0.22%), whereas chronic hypoxia leads to a decrease in sphere formation’s efficiency (0.17%). Radiation seems to play a role only in the acute hypoxic cells, changing the sphere formation efficiency from 0.11% to 0.77% (Figures 4A, B). The size of the spheres decreases under acute and chronic hypoxia, as well as the number of cells involved in forming spheres (Supplementary Figure S2A), however the hypoxic cells have a higher tendency to form clusters, with 4–7 spheres clumping together (Figure 4D). Furthermore, the combination with radiation further reduces the size of hypoxic but not normoxic spheres (*p*-value: 0.000105), and their tendency to form clusters together (Figures 4A, C, E). To investigate the role played by CD133 in the sphere formation and size, we further sorted the cells. In contrast to the whole population of cells, chronic hypoxia significantly increases the sphere formation capacity of LM8 CD133+ cells (∼0.8%) (Figures 5A, B) alone or in combination with radiation (∼0.6%), while for acute hypoxic LM8 CD133+ cells, the exposition to 4 Gy of X-rays is a fundamental condition to increase the sphere formation ability (∼0.63%). However, for CD133+ cells, the sphere size decreases with increased exposure time to hypoxia (Figure 5C), as does the number of cells forming spheres (Supplementary Figure S3A). The number of spheres clustering together increases under acute hypoxia, and even more so in the combination with radiation and chronic hypoxia (Figures 5D, E).

### 3.4 Migration ability of the cells after acute and chronic hypoxia

**FIGURE 6:**
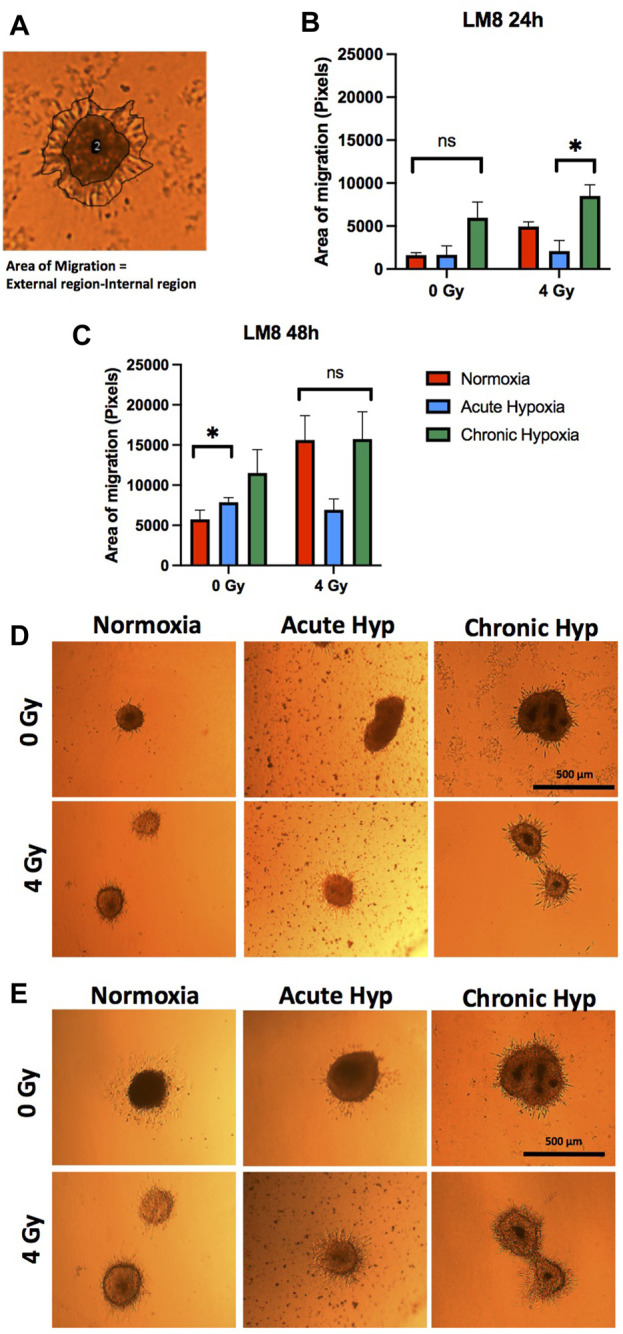
Migration assay of the sphere in collagen. Comparison was made between normoxia, acute hypoxia and chronic hypoxia, with 0 Gy as the control and 4 Gy as the irradiation dose. **(A)** Representative image of migration area analysis (external region–internal region). **(B, D)** The area of migrating cells from the sphere 24 h and **(C, E)** 48 h after embedding. The sphere images (*n* = 3) were analyzed after manual and automatic annotation. Hypoxic cells vs. normoxic control cells. ± SEM, n. s (*p* > 0.05). Unpaired Student’s t-test.

LM8 cells exposed to chronic hypoxia increase the migration ability strongly (from ∼16010 pixels to ∼60,000 pixels of area, after 24 h) (Figures 6A–C ). Normoxic cells increase their migration capacity only after exposure to radiation. No changes have been found after radiation in the cells cultivated in acute or chronic hypoxia (Figures 6B–E). Concerning the migration study of the sorted CD133+ cells, it must be noted that the reoxygenation of the samples during the sorting procedure is not avoidable. We showed that after reoxygenation, there is a substantial decrease in migration ability (Supplementary Figure S4).

### 3.5 CD47 increases under chronic hypoxia

**FIGURE 7:**
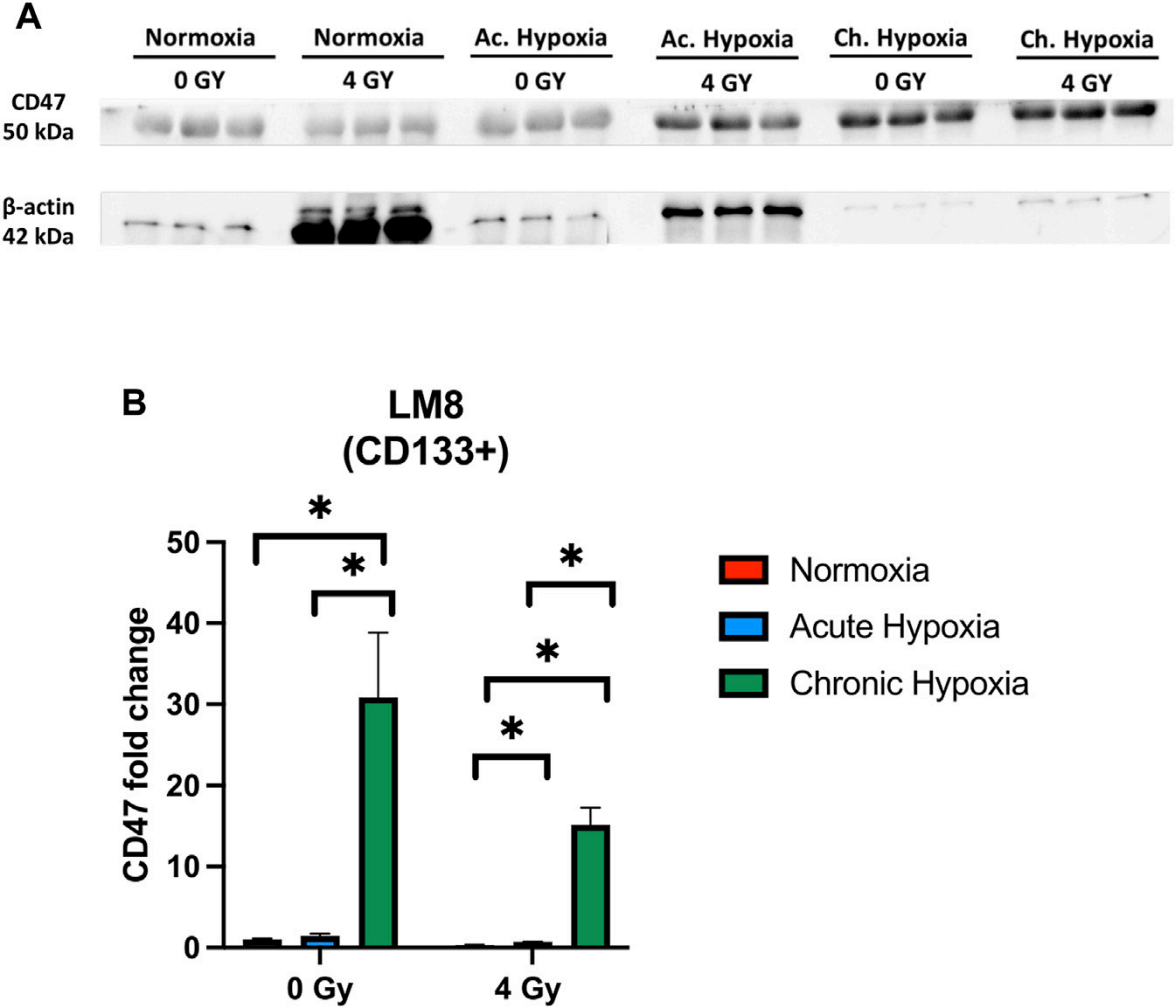
The effects of hypoxia and radiation on CD47 protein expression. **(A)** Representative experiment of Western blot, comparing normoxia, acute hypoxia and chronic hypoxia, with 0 Gy as the control and 4 Gy as the irradiation dose used. **(B)** Mean densitometry of CD47 relative to b-actin is also shown and expressed in arbitrary units. *Hypoxic cells vs. normoxic control cells. ± SEM, *p* < 0.05, unpaired Student’s *t*-test.

After being able to identify a subpopulation of cells with an increased stemness capacity, aggressiveness and migration capability, we further investigated whether these cells express CD47, needed by Circulating Cancer Stem Cells to evade from the immune system control (Yang et al., 2015). We observed a significant increase in the CD47 expression under chronic hypoxia, particularly in the non-irradiated samples (*p*-value: 0.0205), while normoxia and acute hypoxia did not influence CD47 concentration (Figures 7A, B).

## 4 Discussion

More than 90% of tumor-related deaths are caused by tumor relapse and metastasis (Riggio et al., 2021). Not all tumor cells can form metastasis. Only a subpopulation activates the ‘‘invasion-metastasis cascade”: these cells detach from the primary tumor and undergo a morphological change known as Epithelial-to-Mesenchymal transition (EMT), allowing them to intravasate into the blood vessels (Martin et al., 2013; Tinganelli and Durante, 2020). In the blood flow, these circulating tumor cells are subjected to stress, and most of them die because of *anoikis* or because the immune system detects them as not-self and eliminates them (Wang et al., 2018). The CTCs can circulate in the bloodstream as single cells or cell clusters (Hurtado et al., 2023). CTCs clusters are heterogeneous, composed of cells with different phenotypes (Menyailo et al., 2020). The responsible for metastasis formation is a small subset of these cells characterized by the stemness capacity, resistance to *anoikis*, immune evasion ability and higher invasiveness (Yang et al., 2015; Papaccio, 2020). This subset of the CTCs is called Circulating Cancer Stem Cells (CCSCs) (Shiozawa et al., 2013; Tinganelli and Durante, 2020). These cells undergo to a Mesenchymal-to-Epithelial transition (MET), thus extravasating from the blood vessels, disseminating and forming metastasis at distant sites (Banyard and Bielenberg, 2015). They can also be identified by specific markers like CD133 (associated with migration and stemness), TrkB (related to resistance to *anoikis*), and CD47 (a “do not eat me” signal for immune escape) (Geiger and Peeper, 2005; Yang et al., 2015; Helm et al., 2019). Given the low quantity of these cells in the bloodstream, the possibility of select them *in vitro* from the entire population of cancer cells would significantly enhance our understanding of the mechanisms involved in the metastasis formation. In literature is known that hypoxia triggers the expression of CD133 in osteosarcoma cells (Heddleston et al., 2010). However, hypoxia, in these studies, only seems to increase cancer cell stemness (Yun and Lin, 2014; Zhang et al., 2021). There are no studies to verify whether hypoxia can select the subpopulation of cells with a CTCs-like phenotype.

Our study demonstrates that the exposure time to hypoxia influences the cells’ phenotype. Several studies demonstrate that acute hypoxia induces a strong increase of the protein HIF1a (Lin et al., 2011; Masoud and Li, 2015). In contrast, cells exposed to prolonged hypoxia begin to reduce the production of HIF1a protein, and increase the production of HIF2a. The transition from HIF1a to HIF2a marks the shift from acute hypoxia to chronic hypoxia, as these proteins activate distinct gene pathways (Saxena and Jolly, 2019). Furthermore, HIF1a is highly expressed in hematopoietic stem cells, where it activates genes responsible for maintaining a dormant state with low metabolism level (Takubo et al., 2010). In contrast, HIF2a protein activates genes responsible for tumor aggressiveness and invasion (Zhang et al., 2017; Davis et al., 2022).

In this study, we cultivate murine osteosarcoma cells (LM8) in hypoxia. Differently from what is reported in literature, the originality of our study is that we maintained the cells in 1% O_2_ for prolonged time (1 week and 2 weeks), to mimic the hypoxic environment that occurs in tumor. Although we anticipated a decrease in HIF1a after 1 week, our results show that this hypoxic marker not only is still present but also increases after 1 week of hypoxia and it decreases only at 2 weeks. As a result, we categorized the 1-week exposure as “acute hypoxia” and the 2-week exposure as “chronic hypoxia”. Furthermore, we studied the effects of these prolonged hypoxic exposure on the cell’s phenotype.

We demonstrated that acute hypoxia increases CD133 expression and the percentage of cells expressing TrkB in the entire LM8 cell population. However, the overall expression levels of these markers remained low. Interestingly, we expected an increase in the CD133+ sorted cell population but we observed an overall increase in TrkB in CD133+ cells, particularly under hypoxia, where the expression of TrkB shifted from approximately 7% of cells to 40%–50% of cells. These results not only suggest a correlation between CD133 and TrkB markers, but also indicate that hypoxia augments the subpopulation of cells possessing stem-like properties and resistance to *anoikis*. We also found that acute hypoxia enhances the sphere formation capacity of the cells, a characteristic typically associated with cancer stem cells, especially when combined with radiation. In contrast, we observed similar sphere-forming capacities between normoxia and chronic hypoxia. Additionally, with prolonged exposure to hypoxia, we observed a reduction in the size of the spheres, especially when combined with radiation. These findings suggest that hypoxia may induces changes in cell size, enabling them to infiltrate blood vessels and reach distant sites from primary tumor. These characteristics are reminiscent of CTC cells (Douma et al., 2004; Lin et al., 2021a; Chen et al., 2022).

In our study, prolonged exposure to hypoxia decreases the proliferation rate of the cells. We observed smaller spheres in size, but with a tendency to form more clusters, indicating that hypoxia contributes to selecting cells with an aggressive phenotype. However, in the LM8 whole population, we observed that radiation influences the clumping capacity of the spheres, causing them to form fewer clusters. Moreover, in the CD133+ spheres, even though the area of the spheres decreases with an increased exposure to hypoxia, we observed an increased capacity to form clusters under acute hypoxia. This capability is even more enhanced in the combination of chronic hypoxia and radiation. This might indicate that irradiation and prolonged exposure to hypoxia select aggressive cells with a CTC-like phenotype (Li et al., 2016; Ishiguro et al., 2017).

Interestingly, our current results show an increase in cell migration in chronic hypoxia cells but not in acute hypoxia. This phenomenon might be attributed to the cells being in a quiescent state when exposed to acute hypoxia, and their migration capacity is increased after prolonged hypoxia. This might be a hallmark of aggressiveness in circulating cancer stem cells (Fares et al., 2020). As already known, the subpopulation of cells that circulate in bloodstream, known as Circulating Tumor Cells, express CD47, the “do not eat me” signal that allows them to evade detection by the immune system (Lian et al., 2018b; Chen et al., 2022). It is also reported that hypoxia influences the behavior of cells in terms of stemness capacity, migration ability and clustering (Donato et al., 2020). However, there are no studies that investigate whether the duration of hypoxia (1 week or 2 weeks) influences the behavior and aggressiveness of this subpopulation of cells. We investigated whether this subset of aggressive and highly migratory cells expresses CD47 under prolonged hypoxic exposure. Our study demonstrates a significant increase in CD47 expression under chronic hypoxia, indicating that this stressor not only increases cell mobility, ability to form clusters, and decreases sphere size, but also increases the expression of the “do not eat me” signal marker, which is fundamental for the formation of CTC-like cells.

In conclusion, photon irradiation is more likely to be involved in the initial stages of the process, and it becomes particularly significant when combined with acute hypoxia. This combination enhances the cells’ capacity to create spherical structures and clusters, which, in turn, boosts their stem-like characteristics, ultimately giving them a more aggressive phenotype. Moreover, chronic hypoxia becomes relevant in later stages of the metastatic process, conferring to murine osteosarcoma cells, LM8, a Circulating Cancer Stem Cell-like phenotype. This phenotype includes increased migration ability, resistance to *anoikis*, TrkB expression, and immune escape through the expression of the marker CD47. To gain a deeper understanding of the phenotype and the molecular pathways involved in the formation of CCSCs, further *in vivo* studies should be conducted based on this *in vitro* model.

## Data Availability

The raw data supporting the conclusions of this article will be made available by the authors, without undue reservation.

## Supplementary material

SUPPLEMENTARY FIGURE S1
**(A)** Representative experiment of western blot comparing normoxia, 1-week hypoxia and 2-weeks hypoxia.

SUPPLEMENTARY FIGURE S2 Comparison of the total number of cells forming spheres in LM8 full population. **(A)** Bar plot of the comparison between normoxia, acute hypoxia and chronic hypoxia, with 0 Gy as the control and 4 Gy as the irradiation dose used. *hypoxic vs normoxic conditions ± SEM, *P* < 0.05. •Sham controls vs. irradiated ± SEM, *P*< 0.05, unpaired Student’s t-test.

SUPPLEMENTARY FIGURE S3 Comparison of the total number of cells forming spheres in LM8 CD133-positive subpopulation **(A)** Bar plot of the comparison between normoxia, acute hypoxia and chronic hypoxia, with 0 Gy as the control and 4 Gy as the irradiation dose used. *hypoxic vs normoxic conditions ± SEM, *P*< 0.05. •Sham controls vs. irradiated ± SEM, *P* < 0.05. unpaired Student’s *t*-test.

SUPPLEMENTARY FIGURE S4 Migration assay of CD133 sorted LM8 sphere in collagen. Comparison was made between normoxia, acute hypoxia and reoxygenated hypoxia, with 0 Gy as the control, 4 Gy as the irradiation dose used. **(A,C)** The area of migrating cells from the sphere 24 h and **(D, E)** 48 hours after embedding. The sphere images (*n* = 6) were analyzed after manual and automatic annotation. Reoxygenated cells vs normoxic cells. ± SEM, P<0.05; Acute hypoxic cells vs reoxygenated cells. ± SEM, *P* < 0.05. unpaired Student’s *t*-test.
