# Supplementary material for: The role of hypoxia and radiation in developing a CTCs-like phenotype in murine osteosarcoma cells
Source: Front Cell Dev Biol. 2023 Nov 16;11:1222809. doi: 10.3389/fcell.2023.1222809 (PMC10687637; doi:10.3389/fcell.2023.1222809)
Supplement: Supplementary file 1 [file Presentation1.PPTX]

## Slide 1
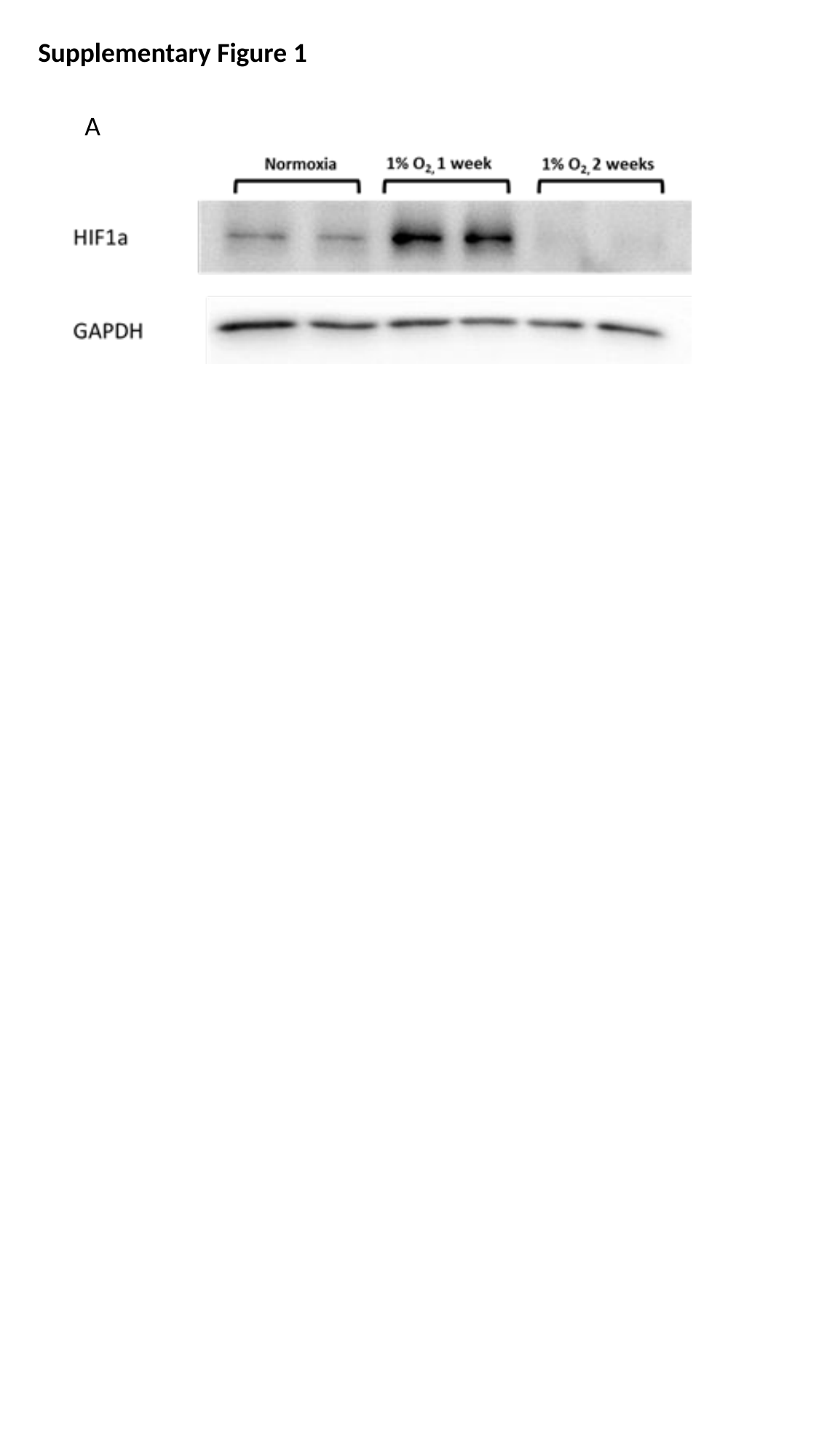

Supplementary Figure 1
A

## Slide 2
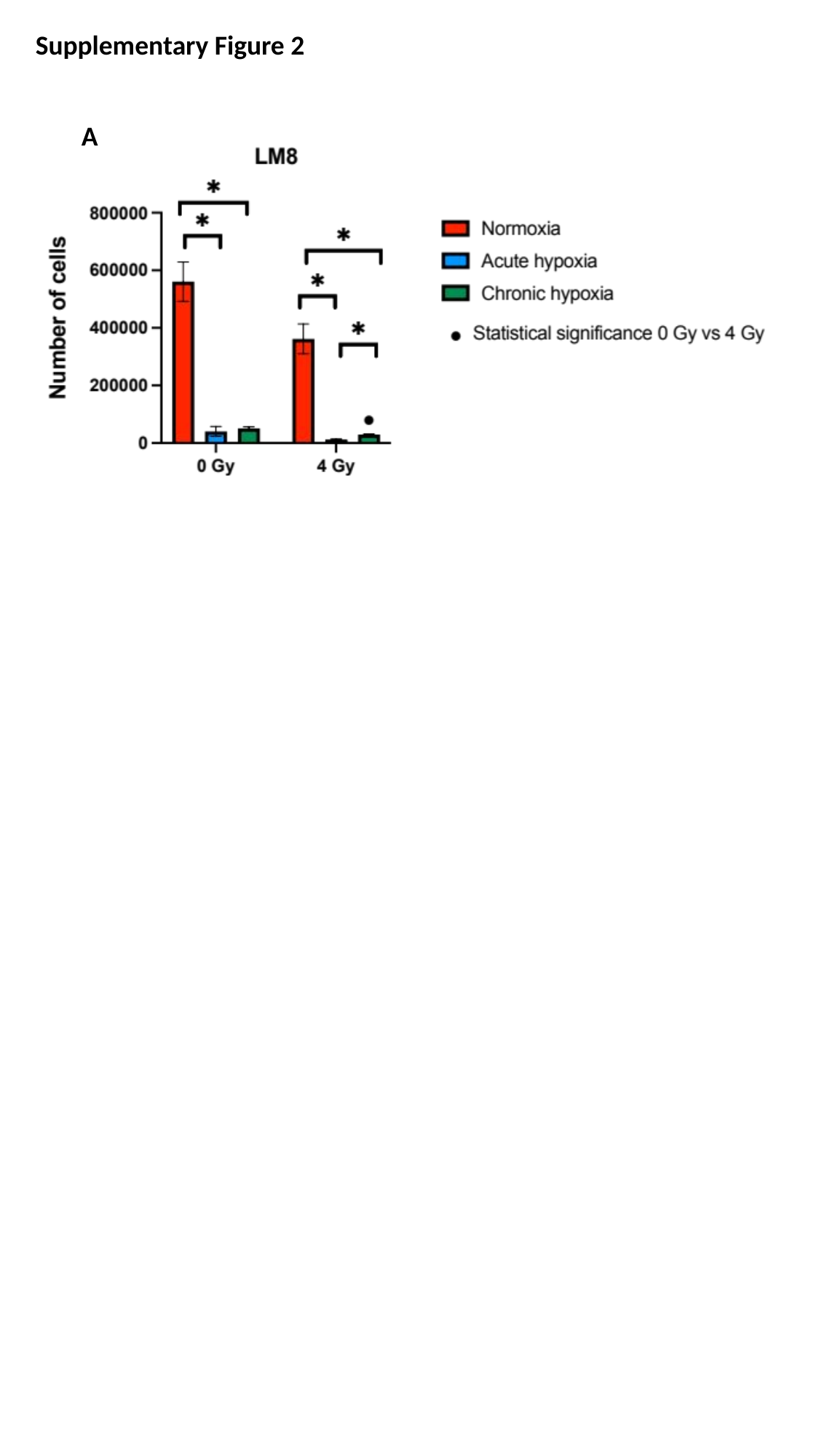

Supplementary Figure 2
A

## Slide 3
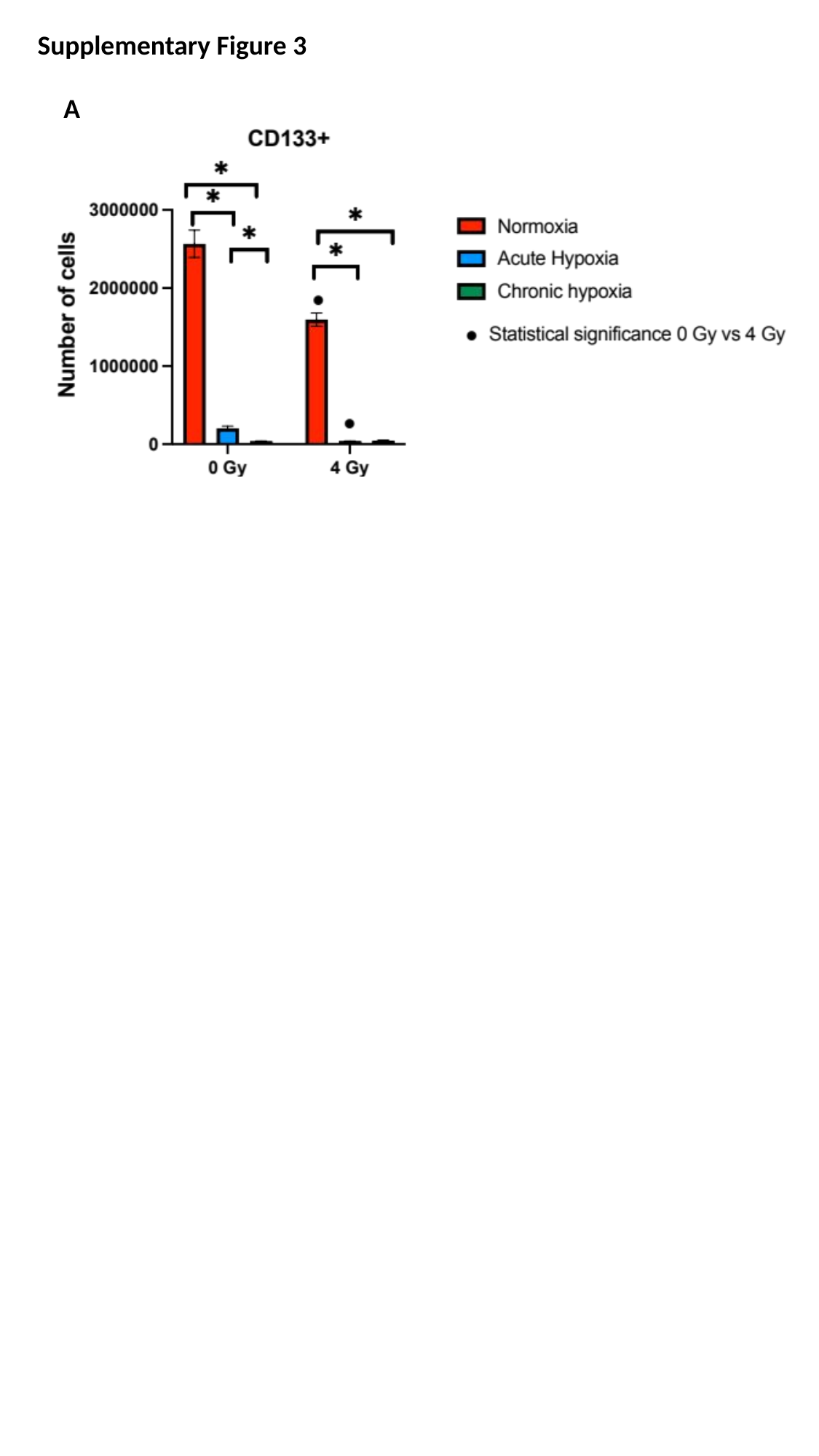

Supplementary Figure 3
A

## Slide 4
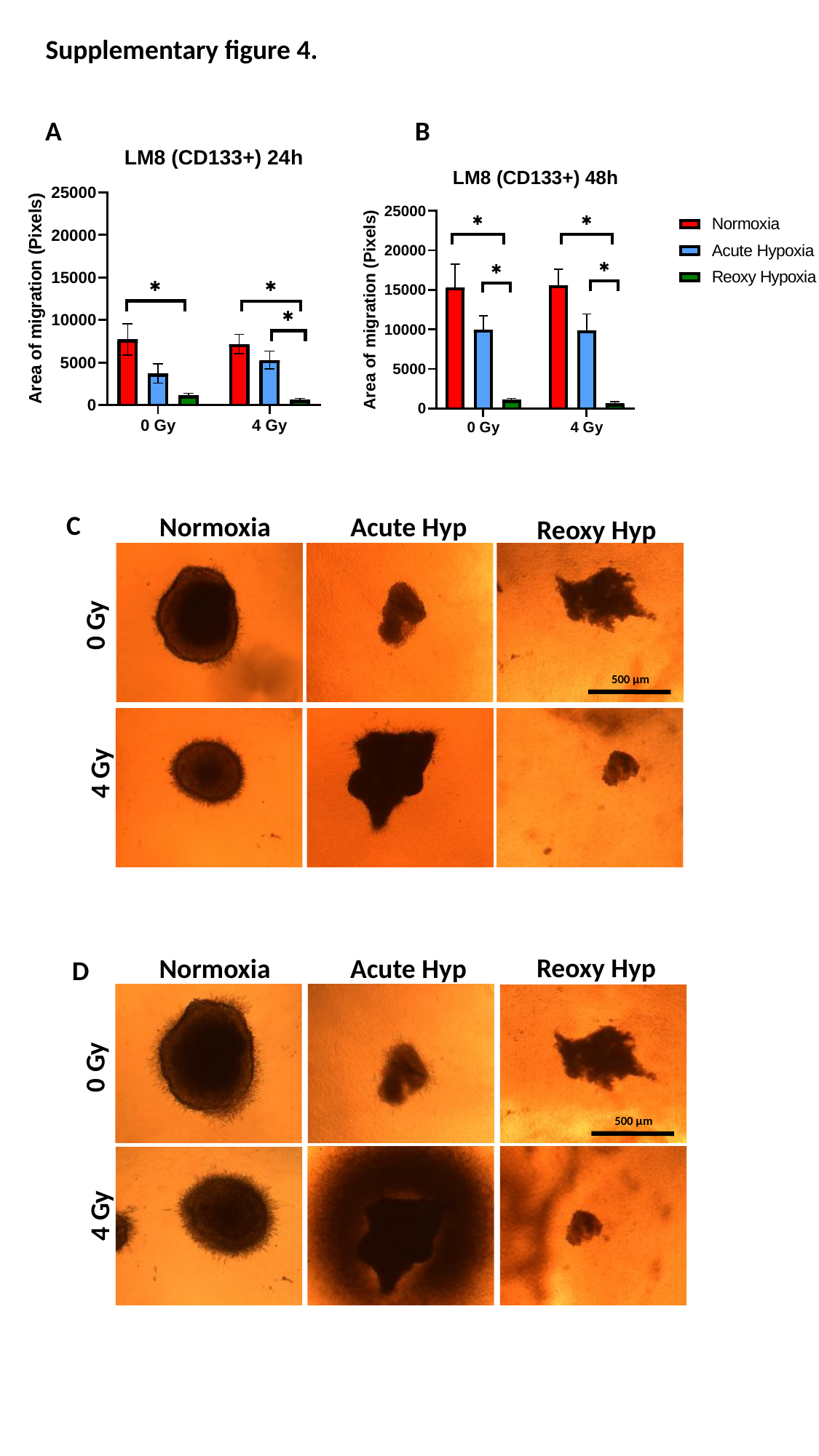

Supplementary figure 4.
A
B
C
Normoxia
Acute Hyp
Reoxy Hyp
0 Gy
500 µm
4 Gy
Reoxy Hyp
Normoxia
Acute Hyp
D
0 Gy
500 µm
4 Gy
